# The Use of ABILHAND-Kids in Children with Unilateral Congenital Below-Elbow Deficiencies and Acquired Amputation: An Italian Cross-Sectional Study

**DOI:** 10.3390/children11080988

**Published:** 2024-08-14

**Authors:** Gessica Della Bella, Luigino Santecchia, Paola Luttazi, Giordana Mariani, Lorenzo Pochiero, Alessandra Lacopo, Caterina Delia, Marco Tofani

**Affiliations:** 1 Management and Diagnostic Innovations & Clinical Pathways Research Area, Neurorehabilitation and Adapted Physical Activity Day Hospital, Bambino Gesù Children’s Hospital, IRCCS, 00165 Rome, Italy; gessica.dellabella@opbg.net (G.D.B.); paola.luttazi@opbg.net (P.L.); giordana.mariani@opbg.net (G.M.); lorenzo.pochiero@opbg.net (L.P.); alessandra.lacopo@opbg.net (A.L.); caterina.delia@opbg.net (C.D.); 2 Orthopedic Department, Hand Surgery and Orthoplastic Service, Bambino Gesù Children’s Hospital, IRCCS, 00165 Rome, Italy; luigino.santecchia@opbg.net; 3 Management and Diagnostic Innovations & Clinical Pathways Research Area, Professional Development, Continuous Education and Research Service, Bambino Gesù Children’s Hospital, IRCCS, 00165 Rome, Italy; 4 Department of Life Sciences, Health and Allied Healthcare Professions, Link Campus University, Via del Casale di San Pio V, 44, 00165 Rome, Italy

**Keywords:** amputation, unilateral below-elbow deficiencies, children, hand function, prosthetics, validation

## Abstract

Congenital or acquired hand differences, including unilateral below-elbow deficiencies, present complex challenges in pediatric rehabilitation. Surgical management and prosthetic provision represent a big challenge to find a good balance for guaranteeing optimal hand function. There is no specific assessment tool for measuring these aspects in the Italian context. The present study investigates the psychometric properties of the ABILHAND-Kids in children with congenital unilateral below-elbow deficiencies and acquired amputation of the upper limb. We measure internal consistency using Cronbach coefficient alpha and the intraclass correlation coefficient (ICC) for measuring test-retest reliability. Differences in hand function in both children with acquired or congenital diseases were also investigated. Participants to the study were 107 (49 F and 58 M) children, with a mean (SD) age of 8.88 (4.25). For test retest reliability, conducted on a sub-sample of 58 children, the ICC was 0.92, while for internal consistency, the Cronbach coefficient alpha was 0.90. We did not find statistically significant differences in scoring (*p* = 0.33) in the use (mean 29.25 SD 6.58) or non-use of a prosthetic device (mean 30.74 SD 7.43), while statistically significant differences were found in hand function (*p* < 0.01) for children who had a congenital impairment (mean 31.87 SD 6.49) and children who had an acquired amputation (mean 27.77 SD 6.60). In conclusion, the ABILHAND-Kids showed good internal consistency and reliability and can capture differences in hand function in children with both congenital and acquired hand disorders.

## 1. Introduction

Congenital hand differences, including unilateral below-elbow deficiencies, present complex challenges in pediatric orthopedics and rehabilitation, affecting approximately 0.2% of all live births [1,2]. Despite advances in medical technology and surgical techniques, there remains a critical need for comprehensive assessment tools and interventions tailored to the unique needs of this population.

The decision-making process regarding amputation versus reconstruction (e.g., hand transplantation, Krukenberg surgery) in children with unilateral below-elbow deficiencies requires careful consideration of various factors, including functional outcomes, cosmetic concerns, psychosocial implications, and long-term quality of life [3,4]. Functional outcomes assessment plays a crucial role in guiding treatment decisions and optimizing rehabilitation strategies [5]. Rehabilitation strategies play a pivotal role in prosthesis acceptance and use; a recent study highlights how a specific home-based rehabilitation protocol can have a positive effect on both cortical reorganization and gross manual dexterity in children with upper-limb reduction deficit [6]. Traditional measures of function, such as strength, range of motion, and sensory perception, provide valuable insights but may not fully capture the complex interplay between impairments and functional limitations in daily life activities [7,8]. Despite progress in children with upper limb deficiencies management, there is a lack of specific outcome measure instruments for this target population [9], while according to the International Classification of Functioning, Disability, and Health (ICF) framework [10], activity limitation and restricted participation should be carefully investigated to guarantee good overall functioning and satisfactory quality of life. The ICF offers a comprehensive model for understanding disability and guiding assessment and intervention strategies in pediatric rehabilitation [11]. However, significant gaps remain in our understanding of the long-term outcomes and optimal management strategies for children with unilateral below-elbow malformation [12]. Children are treated differently in various parts of the world based on different funding, family support, and therapy resources; prosthetic treatment is common for children, although ages for fitting vary among clinics and countries and there are no specific guidelines for assessment, prosthesis prescription, and rehabilitation [13].

The recent literature highlights the importance of patient-centered outcome measures that encompass physical function and psychosocial well-being [14]. Studies have emphasized the need for holistic approaches to assessment and intervention, considering the impact of limb differences on various aspects of children’s lives [15,16]. Furthermore, advancements in prosthetic technology and surgical techniques have expanded treatment options for children with unilateral below-elbow malformation [17,18]. However, challenges persist in achieving optimal functional outcomes and addressing the psychosocial impact of limb differences [19,20].

Functional tests evaluate a child’s “capacity” (what they can do in a controlled environment), while questionnaires assess “performance” (what they actually do in daily life); thus, both dimensions should be measured. Several functional tools exist to assess arm/hand function, but most are not specifically tailored for children with acquired amputations or unilateral below-elbow malformations. A systematic review by Buffart et al. [21] identified only two functional tests (Assisting Hand Assessment [22] and the Unilateral Below Elbow Test [23]) and two questionnaires (ABILHAND-Kids [24] and the Prosthetic Upper Extremity Functional Index (PUEFI) [25,26] as appropriate for evaluating arm/hand function in children with congenital upper limb deficiencies. Among these, ABILHAND-Kids is suitable for children with both unilateral and bilateral impairments. Given that many daily activities require the coordinated use of both hands, assessing bimanual performance provides a more accurate measure of functional limitations in children with unilateral and bilateral congenital hand deformities [27].

ABILHAND-Kids is a Rasch-developed measure of manual ability, defined as the capability to perform daily activities requiring the use of the upper extremities [24]. It consists of 21 items related to bimanual activities, rated by parents based on the difficulty encountered by the child when performing each activity. ABILHAND-Kids was initially developed for children with cerebral palsy (CP) and has been found to be a reliable, valid, and responsive measure for assessing manual ability in children with upper limb impairments [28,29]. The ABILHAND-Kids questionnaire has been utilized as an outcome measure in numerous interventional studies [30,31,32,33] and in various clinical conditions, such as dystonia and choreoathetosis [34], radial deficiency [35], obstetric brachial plexus injury [36], pediatric ischemic stroke [37], and neuromuscular disease [38].

ABILHAND-Kids was originally developed in French and has been successfully translated and validated into several other languages, including Arabic [39], Persian [40], Turkish [38], Brazilian [41], Danish [42], and Ukrainian [43]. Recently, the Italian version of ABILHAND-Kids was translated and demonstrated excellent psychometric properties in a population of children with CP [44]. Having assessment tools specifically applicable to the study population and validated in the language of use is crucial. Therefore, the aim of this study is to validate the Italian version of the ABILHAND-Kids questionnaire for use in clinical practice with a population of children with acquired amputation and unilateral below-elbow deficiencies.

## 2. Materials and Methods

The original version of the ABILHAND-Kids in English underwent translation and cultural adaptation into Italian in a previous study, where it was validated on children with cerebral palsy [44]. In this study, the ABILHAND-Kids-IT underwent testing for validity and reliability within a population of children with acquired amputation or unilateral below-elbow deficiencies. The study was conducted in the Bambino Gesù Childrens’ Hospital IRCCS in Rome, Italy.

### 2.1. Participants

Consistent with consensus-based standards for selecting health measurement instruments, a sample size of 100 patients was deemed appropriate for assessing validity [45]. Similarly, a sample size of at least 50 participants was recommended for conducting a test-retest reliability study [46]. Participants were recruited from the Department of Intensive Neurorehabilitation and Robotics at the Bambino Gesù Children’s Hospital in Rome between April 2022 and May 2023. Inclusion criteria encompassed children aged 6 to 15 years with unilateral below-elbow deficiencies or acquired amputations, accompanied by parents or caregivers fluent in Italian. Exclusion criteria comprised children who had undergone hand surgery in the past three months, bilateral upper limb impairments, or had other associated medical conditions (orthopedic, neurological, or cognitive diseases).

### 2.2. Measurement Tool

The ABILHAND-Kids is a parent-reported outcome measure designed to evaluate the capability of children to perform daily activities using any available strategies [26]. Constructed through the Rasch measurement model, it includes 21 items or manual tasks, each assessed on an ordinal three-point scale: impossible (0 points), difficult (1 point), or easy (2 points). Items not attempted in the past three months are marked with a question mark, unless the activity is deemed impossible for the child, in which case it is scored as impossible. A cumulative score (ranging from 0 to 42 points) is derived by adding the scores for each item. Scores with over 50% missing items are excluded. Lower scores reflect reduced manual ability. The scale confirms unidimensionality [26]. The Italian version of the ABILHAND-Kids [40], utilized in this study, is accessible on the official website http://rssandbox.iescagilly.be/abilhand-kids-downloads.html (accessed on 5 March 2024).

### 2.3. Procedures and Data Analysis

An experienced occupational therapist conducted in-person interviews with parents, supplemented by phone interviews for reliability testing in some cases. Prior to test administration, each parent received a briefing and signed written consent. Descriptive participant data were gathered from clinical records, parental interviews, and medical examinations. Parents were provided with instructional materials to ensure accurate scoring of the ABILHAND-Kids. To assess test-retest reliability, parents were asked to complete the ABILHAND-Kids-IT a second time, 7 to 15 days after the initial evaluation, using an email and web-based data collection approach.

Sociodemographic characteristics and clinical data were summarized using frequency tables, means, and standard deviation (SD). The reliability of the scale was assessed in terms of internal consistency and test-retest reliability. Internal consistency was measured using Cronbach’s alpha, with values above 0.7 considered acceptable [47]. Intraclass correlation coefficients (ICCs) were calculated to estimate test-retest reliability, with interpretation based on predefined criteria [48]. Additionally, the relationship between ABILHAND-Kids mean scores and the use/non-use of prosthetic device and congenital or acquired impairment were explored using *t*-test [49]. Significance was set at *p* < 0.05 95% CI. All statistical analyses were conducted using the IBM Statistical Package for the Social Sciences (SPSS, Version 20.0).

## 3. Results

A total of 107 (49 F and 58 M) participants participated in the study. Their mean (SD) age was 8.88 (4.25) and the majority of the children (*n* = 58) did not use a prosthetic device. For reliability analysis, only 62 parents agreed to be retested. Sample characteristics for both the total sample and the test-retest reliability sub-sample are summarized in Table 1.

The ABILHAND-kids was administered to the whole sample, obtaining a mean (SD) total score of 30.80 (6.97), while the Cronbach coefficient alpha for the whole scale was 0.90. In Table 2 are the synthetized means (SD) score for each item and the values for the item-total statistics.

**Figure 1:**
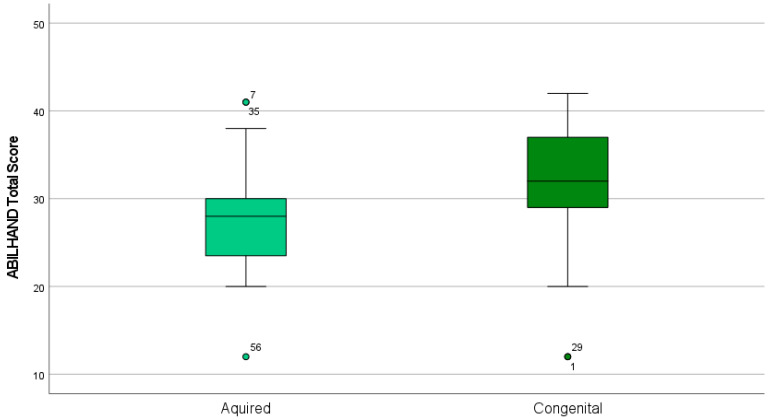
Mean difference (*p* < 0.01) in ABILHAND-kids total score according to acquired or congenital impairment.

Concerning test-retest reliability, our analysis found an excellent value of ICC 0.92 (lower bound 0.87–upper bound 0.96). We also analyzed differences in the ABILHAND-Kids score according to acquired or congenital impairments and the use or non-use of a prosthetic device; we did not find statistically significant differences in scoring (*p* = 0.33) in the use (mean 29.25 SD 6.58) or non-use of a prosthetic device (mean 30.74 SD 7.43), while statistically significant differences were find in hand function (*p* < 0.01) for children who had a congenital impairment (mean 31.87 SD 6.49) and children who had an acquired amputation (mean 27.77 SD 6.60). The results for children who had acquired or congenital impairment are graphically presented in Figure 1.

## 4. Discussion

The present study sought to validate the ABILHAND-Kids in a population of children with congenital amputations or unilateral below-elbow deficiencies and found good psychometric properties in terms of internal consistency and reliability.

Our analyses revealed very good internal consistency, with a Cronbach’s alpha coefficient (0.90), consistent with the Italian validation study on children with CP (0.98) [44], as well as other validation studies, such as those conducted in Turkey (0.94) [38], Brazil (0.99) [41], Iran (0.96) [40], and Ukraine (0.95) [43]. This finding confirms the homogeneity of the scale and the relatedness of its items. Regarding test-retest reliability, our findings confirm the good stability of the scale with an ICC of 0.92, consistent with the Italian version (0.99) and also with studies conducted in Saudi Arabia (0.98) [39], Iran (0.96) [40], Brazil (0.97) [41], Turkey (0.98) [38], Denmark (0.97) [42], and Belgium (0.91) [24]. Therefore, ABILHAND-Kids can be considered a reliable tool when used to measure hand function in children with acquired amputations or unilateral below-elbow deficiencies after 7–15 days from the initial administration.

However, it is important to highlight that out of the entire sample (*n* = 107), only 62 parents opted to participate in the retest analysis, resulting in a significant loss of data for children. This could possibly be attributed to the approach used for test-retest administration. Instead of conducting phone or in-person interviews, we chose to send an email with the ABILHAND-Kids to parents who agreed to participate. While we believed that a web-based approach for data collection might be more convenient for parents, it ultimately resulted in a low response rate, as reported by Koh and colleagues in a different setting with various age groups and health conditions [50]. Nevertheless, according to the COSMIN guidelines, our sample size for test-retest analysis was considered sufficient, and therefore, the results can be deemed appropriate.

Interesting findings emerged from the analysis of items and total scores of the ABILHAND-Kids. Firstly, we found that for children with congenital amputation or unilateral below-elbow deficiencies, the most challenging items were Q18 (Buttoning up trousers) with a mean SD score of 0.88 (0.58), Q20 (Zipping up a jacket) with a mean SD score of 0.87 (0.82), Q6 (Rolling up a sleeve of a sweater) with a mean SD score of 1.03 (0.61), and Q13 (Buttoning up a shirt/sweater) with a mean SD score of 1.04 (0.72). Dressing is a fundamental aspect of daily life that can pose significant challenges for children with amputations. The loss of a limb or deficiencies of the upper limb can impact their ability to independently dress and undress. For example, children with upper limb amputations may struggle to manipulate small fasteners or reach behind their backs to secure clothing items. They may need to develop alternative methods, such as using one-handed techniques or adaptive tools like button hooks, zipper pulls, or Velcro closures. Additionally, selecting clothing with features like larger buttons, elastic waistbands, or magnetic closures can facilitate independent dressing [51,52]. Beyond the physical aspects, dressing tasks can also affect a child’s confidence, self-esteem, and sense of autonomy; frustration or difficulty in completing these tasks independently may lead to feelings of dependence or inadequacy. Therefore, it is essential to provide encouragement, support, and opportunities for skill development to empower children with amputations to overcome these challenges and achieve greater autonomy in dressing and other activities of daily living [53].

Items that obtained higher scores include Q16 (Putting on a hat) (mean 1.92, SD 0.27), Q8 (Putting on a backpack) (mean 1.88, SD 0.43), Q15 (Switching on a bedside lamp) (mean 1.92, SD 0.26), and Q8 (Taking off a t-shirt) (mean 1.80, SD 0.40). This was expected as all these actions can be easily managed with one hand or alternative strategies. Surprisingly, high scores were also observed for Q7 (Sharpening a pencil) and Q11 (Unscrewing a bottle cap), with mean SD scores of 1.62 (0.56) and 1.61 (0.57), respectively. Despite these actions requiring good coordination of both upper limbs, individuals with amputations or unilateral below-elbow deficiencies can employ several strategies to complete the tasks, such as forearm stabilization or grasping with the elbow or trunk [54,55,56]. The ABILHAND-Kids requires parents to rate hand function and performance in daily life, irrespective of the strategies used by their children. To better understand this issue, the use of different assessment tools is suggested, such as the Unilateral Below Elbow Test [23], which can provide informative data on both task completion and methods used.

Concerning qualitative analysis of differences in acquired or congenital deficiencies, boxplots highlighting the median values for children with acquired deficiencies showed a left-skewed distribution; 50% of children received a score between 38 and 38, while for children with congenital deficiencies, a right-skewed distribution was observed (50% of children obtained a score between 20 and 34). In children with congenital deficiencies, high variability in IQR was also observed. This can be explained by the different adaptive strategies that children put in place over time. In fact, statistically significant differences in ABILHAND-Kids scores (*p* < 0.01) were found between children with congenital impairments (mean 31.87, SD 6.49) and children with acquired amputations (mean 27.77, SD 6.60). This can be explained by the fact that hand use shapes brain organization [57], and children with congenital deficiencies may develop better compensatory strategies than those with acquired amputations, who require time to reorganize motor schemes for hand function, including the use of a prosthetic device. Neurophysiological embodiment of artificial limbs depends on prosthesis usage in everyday life, and prosthesis usage also shapes large-scale brain reorganization [58]. Timing of using a prosthetic device may also influence acceptance; studies show that children who began wearing their prosthesis before the age of 2 or within 6 months after amputation were less likely to reject their prosthesis [59], and, therefore, it seems crucial to investigate manual motor functions according to the timing of amputation in acquired cases and the timing of initial prosthetic use in congenital differences.

## 5. Limitations

Despite these encouraging and informative results, the present study has several limitations. Firstly, differences were found between children with acquired amputations and congenital deficiencies, but to better understand the mechanisms influencing hand function, additional information is needed, such as the timing of injuries and initial prosthetic fitting. It is worth noting that early upper-limb prosthetic use, frequency of use, and everyday context can impact brain reorganization and hand function [60]. Further studies should consider this aspect, analyzing time of utilization of prosthetic device and the overall type of prosthetic device used (e.g., aesthetic, mechanic, myoelectric prostheses), and by recruiting a larger sample, a multivariate logistic regression analysis can be performed to determine which variables better inform outcomes. A second limitation may be the lack of differentiation in the type of prosthetic device used, whereas having information on aesthetic, functional, or myoelectric devices might explain why differences in scoring were not found in the use or non-use of a prosthetic device (*p* = 0.33). Other limitations concern the psychometric properties, such as the lack of use of Rasch model analysis, as performed in other validation studies. Furthermore, the concurrent validity of the ABILHAND-Kids was not analyzed; although this represents an important psychometric measure when validating a tool, to the best of our knowledge, there is no similar assessment tool already validated for the Italian context. We, therefore, recommend that in the future, ABILHAND-Kids scores could be compared with another outcome measure to assess their level of correlation, as well as measuring other psychometric measures, such as responsiveness and minimum important difference, that can inform clinicians and researchers on how ABILHAND-Kids measurements change over time in this target population.

## 6. Conclusions

In conclusion, the ABILHAND-Kids was found to be a reliable tool for measuring hand function in children with acquired amputations and unilateral below-elbow deficiencies aged between 5 and 18 years

## Figures and Tables

**Table 1:** Socio-demographic and clinical characteristics of the sample (total 20).

	Total	Test-Retest
**Gender**	**N (%)**	**N (%)**
Female	49 (45.8)	27 (43.6)
Male	58 (54.2)	35 (56.4)
**Level of Education**	**N (%)**	**N (%)**
Kindergarten	23 (21.5)	11 (17.7)
Primary school	50 (46.7)	44 (70.9)
Secondary school	12 (11.2)	5 (8.1)
High School	22 (20.6)	2 (3.2)
**Age**	**Mean (SD)**	**Mean (SD)**
Used prosthetic device	8.08 (3.89)	9.12 (3.59)
Do not use prosthetic device	8.94 (4.57)	10.02 (2.36)
Total	8.88 (4.25)	9.73 (3.64)
**Prosthetic Device**	**N (%)**	**N (%)**
Yes	58 (54.21)	32 (51,2)
No	49 (45.79)	30 (48.4)
**Level of Amputation/Deficiency**	**N (%)**	**N (%)**
Fingers	11 (10.3)	4 (6.5)
Hand	34 (31.8)	17 (27.4)
Wrist	12 (12.1)	5 (8.1)
Trans-radial	49 (45.8)	36 (58.1)
**Type of Amputation/Deficiency**	**N (%)**	**N (%)**
Acquired	46 (42.9)	25 (40.2)
Congenital	61 (57.1)	37 (59.8)

**Table 2:** Mean and SD score and Cronbach’s coefficient alpha for each item of the ABILHAND-KIDS.

Item	Description	Mean (SD)	Scale Mean If Item Deleted	Scale Variance If Item Deleted	Corrected Item-Total Correlation	Cronbach’s Alpha If Item Deleted
1	Opening a jar of jam	1.39 (0.56)	29.41	45.578	0.354	0.902
2	Putting on a backpack/schoolbag	1.88 (0.43)	28.92	45.647	0.477	0.899
3	Opening the cap of a toothpaste tube	1.79 (0.41)	29.01	46.893	0.276	0.903
4	Unwrapping a chocolate bar	1.26 (0.61)	29.54	42.678	0.687	0.894
5	Washing the upper-body	1.72 (0.53)	29.08	44.607	0.524	0.898
6	Rolling-up a sleeve of a sweater	1.03 (0.61)	29.78	42.123	0.772	0.891
7	Sharpening a pencil	1.62 (0.56)	29.18	44.792	0.463	0.900
8	Taking off a T-shirt	1.80 (0.40)	29.00	45.733	0.502	0.899
9	Squeezing toothpaste onto a toothbrush	1.51(0.64)	29.29	43.142	0.599	0.896
10	Opening a bread box	1.30 (0.56)	29.50	43.987	0.573	0.897
11	Unscrewing a bottle cap	1.61 (0.56)	29.20	47.014	0.164	0.907
12	Zipping-up trousers	1.18 (0.76)	29.62	40.586	0.768	0.890
13	Buttoning up a shirt/sweater	1.04 (0.72)	29.76	41.676	0.690	0.893
14	Filling a glass with water	1.88 (0.32)	28.92	46.527	0.446	0.900
15	Switching on a bedside lamp	1.92 (0.26)	28.88	47.359	0.316	0.902
16	Putting on a hat	1.92 (0.27)	28.88	48.612	−0.019	0.906
17	Fastening the snap of a jacket	1.18 (0.81)	29.62	40.426	0.729	0.892
18	Buttoning up trousers	0.88 (0.58)	29.92	42,927	0.693	0.894
19	Opening a bag of chips	1.24 (0.60)	29.57	43.129	0.640	0.895
20	Zipping-up a jacket	0.87 (0.82)	29.93	39.769	0.788	0.890
21	Taking a coin out of a pocket	1.76 (0.42)	29.04	47.292	0.193	0.905

## Data Availability

Data are available from the corresponding author upon reasonable request.

## References

[1] Ezaki M., Oishi S.N., Azar F.M., Beaty J.H., Canale S.T. (2017). Congenital hand anomalies. Campbell’s Operative Ortho-Paedics.

[2] Tonkin M.A. (2015). Congenital hand differences. J. Hand Surg. Eur. Vol..

[3] Al-Qattan M.M. (2009). Classification of congenital limb deficiencies. J. Hand Surg. Eur. Vol..

[4] Goldfarb C.A., Wall L.B., Bohn D.C., Moen P., Van Heest A.E. (2015). Epidemiology of congenital upper limb anomalies in a midwest United States population: An assessment using the Oberg, Manske, and Tonkin classification. J. Hand Surg..

[5] United Nations Children’s Fund (UNICEF) (2013). The State of the World’s Children 2013: Children with Disabilities.

[6] A Borrell J., Manattu A.K., Copeland C., Fraser K., D’ovidio A., Granatowicz Z., Delgado L., Zuniga J.M. (2024). Prosthetic home intervention induces cortical plasticity in paediatrics with congenital limb reduction. Brain Commun..

[7] Burger H., Brezovar D., Marinček Č. (2004). Comparison of clinical test and questionnaires for the evaluation of upper limb prosthetic use in children. Disabil. Rehabil..

[8] Dillingham T.R., Pezzin L.E., Mackenzie E.J. (2002). Limb Amputation and Limb Deficiency: Epidemiology and Recent Trends in the United States. South. Med. J..

[9] Koenig K.D., Hall M.J., Gormley C., Kaleta M., Munger M., Laine J., Morgan S.J. (2024). Clinical outcomes measurement in pediatric lower limb prosthetics: A scoping review. J. Pediatr. Rehabil. Med..

[10] The World Health Organization (2001). International Classification of Functioning, Disability, and Health (ICF).

[11] Tofani M., Mustari M., Tiozzo E., Dall’oglio I., Morelli D., Gawronski O., Salata M., Cantonetti L., Castelli E., Di Lallo D. (2023). The development of the International Classification of Functioning, Disability and Health for Child and Youth (ICF-CY) Core Sets: A systematic review. Disabil. Rehabil..

[12] Meurs M., Maathuis C.G.B., Lucas C., Hadders-Algra M., Van Der Sluis C.K. (2006). Prescription of the first prosthesis and later use in children with congenital unilateral upper limb deficiency: A systematic review. Prosthet. Orthot. Int..

[13] Hill W.B., Hermansson L.M. (2023). Treatment for Children with Upper-Limb Differences in Various Parts of the World: Preliminary Findings. JPO J. Prosthet. Orthot..

[14] Michielsen A., Van Wijk I., Ketelaar M. (2010). Participation and quality of life in children and adolescents with congenital limb deficiencies: A narrative review. Prosthet. Orthot. Int..

[15] Davids J.R., Wagner L.V., Meyer L.C., Blackhurst D.W. (2006). Prosthetic management of children with unilateral congenital below-elbow deficiency. JBJS.

[16] Paracuollo M., Novelli C., Proserpio G., Young K., Pajardi G. (2023). Progressive Bone Distraction Lengthening in the Treatment of Congenital Malformations of the Upper Limb. Pediatric Hand Surgery.

[17] Mendenhall S.D., Levy T.J., Amaral S., Chang B., Levin L.S. (2023). Hand Transplantation in Children. Pediatric Hand Surgery.

[18] James M.A., Bagley A.M., Brasington K., Lutz C., McConnell S., Molitor F. (2006). Impact of prostheses on function and quality of life for children with unilateral congenital below-the-elbow deficiency. JBJS.

[19] Battraw M.A., Fitzgerald J., James M.A., Bagley A.M., Joiner W.M., Schofield J.S. (2024). Understanding the capacity of children with congenital unilateral below-elbow deficiency to actuate their affected muscles. Sci. Rep..

[20] Aaron D.H., Jansen C.W. (2003). Development of the Functional Dexterity Test (FDT): Construction, validity, reliability, and normative data. J. Hand Ther..

[21] Buffart L.M., Roebroeck M.E., Pesch-Batenburg J.M., Janssen W.G., Stam H.J. (2006). Assessment of arm/hand functioning in children with a congenital transverse or longitudinal reduction deficiency of the upper limb. Disabil. Rehabil..

[22] Krumlinde-Sundholm L., Eliasson A.-C. (2003). Development of the Assisting Hand Assessment: A Rasch-built Measure intended for Children with Unilateral Upper Limb Impairments. Scand. J. Occup. Ther..

[23] Bagley A.M., Molitor F., Wagner L.V., Tomhave O.W., James M.A. (2006). The Unilateral Below Elbow Test: A function test for children with unilateral congenital below elbow deficiency. Dev. Med. Child. Neurol..

[24] Arnould C., Penta M., Renders A., Thonnard J.L. (2004). ABILHAND-Kids: A measure of manual ability in children with cerebral palsy. Neurology.

[25] Ardon M.S., Janssen W.G., Hovius S.E., Stam H.J., Murawska M., Roebroeck M.E., Selles R.W. (2014). Relationships among manual body functions, manual capacity, and bimanual performance using the prosthetic upper ex-tremity functional index in children with congenital hand differences. Phys. Ther..

[26] Wright F.V., Hubbard S., Jutai J., Naumann S. (2001). The Prosthetic Upper Extremity Functional Index: Development and reliability testing of a new functional status questionnaire for children who use upper extremity prostheses. J. Hand Ther..

[27] Klingels K., Jaspers E., Van de Winckel A., De Cock P., Molenaers G., Feys H. (2010). A systematic review of arm activity measures for children with hemiple- gic cerebral palsy. Clin. Rehabil..

[28] Wagner L.V., Davids J.R. (2012). Assessment tools and classification systems used for the upper extremity in children with cerebral palsy. Clin. Orthop. Relat. Res..

[29] Gilmore R., Sakzewski L., Boyd R. (2010). Upper limb activity measures for 5- to 16-year-old children with congenital hemiplegia: A systematic review. Dev. Med. Child. Neurol..

[30] Geerdink Y., Aarts P., van der Burg J., Steenbergen B., Geurts A. (2015). Intensive upper limb intervention with self-management training is feasible and promising for older children and adolescents with unilateral cerebral palsy. Res. Dev. Disabil..

[31] Acar G., Altun G.P., Yurdalan S., Polat M.G. (2016). Efficacy of neurodevelopmental treatment combined with the Nintendo^®^ Wii in patients with cerebral palsy. J. Phys. Ther. Sci..

[32] Bruchez R., Gygax M.J., Roches S., Fluss J., Jacquier D., Ballabeni P., Grunt S., Newman C.J. (2016). Mirror therapy in children with hemiparesis: A randomized observer–blinded trial. Dev. Med. Child. Neurol..

[33] Speth L., Janssen-Potten Y., Leffers P., Rameckers E., Defesche A., Winkens B., Becher J., Smeets R., Vles H. (2015). Effects of botulinum toxin A and/or bimanual task-oriented therapy on upper extremity impairments in unilateral cerebral palsy: An explorative study. Eur. J. Paediatr. Neurol..

[34] Monbaliu E., de Cock P., Ortibus E., Heyrman L., Klingels K., Feys H. (2016). Clinical patterns of dystonia and choreoathetosis in participants with dyskinetic cerebral palsy. Dev. Med. Child. Neurol..

[35] Buffart L.M., Roebroeck M.E., Janssen W.G., Hoekstra A., Hovius S.E., Stam H.J. (2007). Comparison of instruments to assess hand function in children with radius deficiencies. J. Hand Surg..

[36] Spaargaren E., Ahmed J., van Ouwerkerk W.J., de Groot V., Beckerman H. (2011). Aspects of activities and participation of 7–8 year-old children with an obstetric brachial plexus injury. Eur. J. Paediatr. Neurol..

[37] Kornfeld S., Studer M., Winkelbeiner S., Regenyi M., Boltshauser E., Steinlin M. (2017). Quality of life after paediatric ischaemic stroke. Dev. Med. Child Neurol..

[38] Öksüz Ç., Alemdaroglu I., Kilinç M., Abaoğlu H., Demirci C., Karahan S., Yilmaz O., Yildirim S.A. (2017). Reliability and validity of the Turkish version of ABILHAND-Kids’ questionnaire in a group of patients with neuromuscular disorders. Physiother. Theory Pr..

[39] Alnahdi A.H., Alhusaini A.A., Alshami A., Yousef B., Melam G. (2020). Cross-cultural adaptation and measurement properties of the Arabic version of the ABILHAND-Kids scale. Disabil. Rehabil..

[40] Mohammadkhani-Pordanjani E., Arnould C., Raji P., Ansari N.N., Hasson S. (2020). Validity and reliability of the Persian ABILHAND-Kids in a sample of Iranian children with cerebral palsy. Disabil. Rehabil..

[41] Kamonseki D.H., Cedin L., Clemente A.F., Peixoto B.D.O., Zamunér A.R. (2017). Translation, cross-cultural adaptation and validation of the ABILHAND-Kids for the Brazilian Portuguese. Fisioter. Pesqui..

[42] Hansen A., Poulsen H.S., Kristensen H.K., Lauridsen H.H. (2020). Danish translation, adaptation and validation of the ABILHAND-Kids questionnaire for children with cerebral palsy. Disabil. Rehabil..

[43] Hasiuk M.B., Arnould C., Kushnir A.D., Matiushenko O.A., Kachmar O.O. (2021). Cross-cultural adaptation and validation of the Ukrainian version of the ABILHAND-Kids questionnaire. Disabil. Rehabil..

[44] Tofani M., Blasetti G., Lucibello L., Berardi A., Galeoto G., Sabbadini M., Santecchia L., Castelli E. (2021). An Italian Validation of ABILHAND-Kids for Children With Cerebral Palsy. Percept. Mot. Ski..

[45] Mokkink L.B., Terwee C.B., Knol D.L., Stratford P.W., Alonso J., Patrick D.L., Bouter L.M., De Vet H.C. (2010). The COSMIN checklist for evaluating the methodological quality of studies on measurement properties: A clarification of its content. BMC Med. Res. Methodol..

[46] De Vet H.C., Terwee C.B., Mokkink L.B., Knol D.L. (2011). Measurement [49] Med.

[47] Nunnally J.C. (1979). Psychometric theory. Psychometric Theory.

[48] Lexell J.E., Downham D.Y. (2005). How to Assess the Reliability of Measurements in Rehabilitation. Am. J. Phys. Med. Rehabil..

[49] Terwee C.B., Bot S.D., de Boer M.R., van der Windt D.A.W.M., Knol D.L., Dekker J., Bouter L.M., de Vet H.C.W. (2007). Quality criteria were proposed for measurement properties of health status questionnaires. J. Clin. Epidemiol..

[50] Koh Y.L.E., Lua Y.H.A., Hong L., Bong H.S.S., Yeo L.S.J., Tsang L.P.M., Ong K.Z., Wong S.W.S., Tan N.C. (2016). Using a web-based approach to assess test–retest reliability of the “hypertension self-care profile” tool in an Asian population: A validation study. Medicine.

[51] Adams K. (2016). Adapting clothing for children with limb loss. Prosthet. Orthot. Int..

[52] Han S.Y., Choi H.S., Lee D.G., Lee Y.H. (2019). Clothing Adaptation Strategies for Children with Limb Differences. Text. Res. J..

[53] Gallagher P., MacLachlan M., McAdam J. (2001). Positive experiences of social support and integration in an Irish limb-loss group. Disabil. Rehabil..

[54] Touillet A., Gouzien A., Badin M., Herbe P., Martinet N., Jarrassé N., Roby-Brami A. (2022). Kinematic analysis of impairments and compensatory motor behavior during prosthetic grasping in below-elbow amputees. PLoS ONE.

[55] Battraw M.A., Fitzgerald J., Joiner W.M., James M.A., Bagley A.M., Schofield J.S. (2022). A review of upper limb pediatric prostheses and perspectives on future advancements. Prosthet. Orthot. Int..

[56] Huizing K., Reinders-Messelink H., Maathuis C., Hadders-Algra M., Van Der Sluis C.K. (2010). Age at first prosthetic fitting and later functional outcome in children and young adults with unilateral congenital below-elbow deficiency: A cross-sectional study. Prosthet. Orthot. Int..

[57] Ejaz N., Hamada M., Diedrichsen J. (2015). Hand use predicts the structure of representations in sensorimotor cortex. Nat. Neurosci..

[58] Van den Heiligenberg F.M., Orlov T., Macdonald S.N., Duff E.P., Slater D.H., Beckmann C.F., Johansen-Berg H., Culham J.C., Makin T.R. (2018). Artificial limb representation in amputees. Brain.

[59] Sinskey Y.L., Spires M.C. (2024). Prostheses and Rehabilitation Principles in Pediatric Limb Deficiency. Phys. Med. Rehabil. Clin. North. Am..

[60] Peterson J.K.M., Prigge P.C. (2020). Early upper-limb prosthetic fitting and brain development: Considerations for success. JPO J. Prosthet. Orthot..

